# Contrast Sensitivity Loss in Patients With Posttreatment Lyme Disease

**DOI:** 10.1167/tvst.10.3.27

**Published:** 2021-03-24

**Authors:** Alison W. Rebman, Ting Yang, John N. Aucott, Erica A. Mihm, Sheila K. West

**Affiliations:** 1 Lyme Disease Research Center, Division of Rheumatology, Department of Medicine, Johns Hopkins University School of Medicine, Baltimore, MD, USA; 2 Wilmer Eye Institute, Johns Hopkins University School of Medicine, Baltimore, MD, USA

**Keywords:** lyme disease, posttreatment lyme disease, contrast sensitivity, cognitive impairment, neurologic deficit

## Abstract

**Purpose:**

Posttreatment Lyme disease (PTLD) is marked by neurologic symptoms, cognitive impairment, and significant symptom burden, including fatigue and ocular complaints. The purpose of this study was to determine whether contrast sensitivity (CS) is altered in patients with PTLD compared with healthy controls and, second, whether CS is associated with cognitive and/or neurologic deficits.

**Methods:**

CS was measured using a Pelli–Robson chart with forced-choice procedures, and the total number of letters read was recorded for each eye. CS impairment was defined for age <60 years as logCS of 1.80 (36 letters or fewer) and for those age ≥60 years as logCS of 1.65 (33 letters or fewer). Participants self-administered a questionnaire to assess presence of ocular symptoms and underwent a neurologic exam and battery of neurocognitive tests.

**Results:**

CS impairment was associated with an increased odds of being in the PTLD group that was 2.6 times as high as those without CS impairment (odds ratio, 2.6; 95% confidence interval, 1.3–5.2). Neither cases nor controls had significant distance acuity impairment. CS impairment was not associated with any of the ocular complaints in cases but was borderline associated with neurologic abnormalities and cognitive impairment.

**Conclusions:**

CS impairment in patients with PTLD is linked to signs of cognitive and neurologic impairment and may be a marker of illness severity.

**Translational Relevance:**

Further investigation into the value of testing CS impairment in PTLD cases is warranted, especially if it is an indicator of cognitive or neurologic manifestations.

## Introduction

Lyme disease, caused by infection with *Borrelia burgdorferi,* is the most common tick-borne disease in temperate regions of the Northern Hemisphere.^1^ The Centers for Disease Control and Prevention (CDC) estimates approximately 300,000 new infections occur annually in the United States.^2^ The number of cases has increased in recent decades due in part to climate change, shifting land use patterns, and the relative abundance and distribution of reservoir hosts.^3^

Lyme disease often initially presents with a cutaneous lesion, erythema migrans, at the site of the bite of an infected tick, with or without other concurrent signs of infection.^4^ If untreated, *B. burgdorferi* may then disseminate from the site of skin inoculation to other areas of the skin, as well as the musculoskeletal, cardiac, and neurologic systems.^5^ Ocular symptoms, including photophobia, conjunctivitis, and periorbital edema, have been described in up to 11% of patients in acute infections.^6^ Antibiotic treatment for Lyme disease generally resolves objective signs of infection for most patients.^7^ However, a subset of treated patients develops a chronic illness of persistent or recurrent symptoms following treatment.^8^^,^^9^ The most prominent of these symptoms include fatigue, widespread musculoskeletal pain, and cognitive difficulties, but patients also report a range of neurologic, sleep, ocular, mood, and other symptoms.^10^^–^^12^ A standardized, highly specific definition for posttreatment Lyme disease (PTLD) has been operationalized to identify these patients.^7^^,^^10^^,^^13^

Testing of contrast sensitivity (CS) has been proposed as a means to aid diagnosis, identify neurologic effects, and assess treatment response among patients with PTLD.^14^ CS is impaired in a variety of ocular conditions such as cataract and retinal degeneration, as well as in neurologic diseases, which commonly manifest with optic neuritis, such as multiple sclerosis (MS).^15^^–^^19^ In addition to possibly reflecting ocular structural changes, CS loss may also indicate specific or nonspecific deficits in neurologic and/or cognitive function.^17^^,^^20^^,^^21^

Whether CS is depressed in patients with PTLD or is associated with ocular complaints and/or objective neurologic or neurocognitive findings is not known. This study was able to test CS in a well-characterized cohort of patients with PTLD and controls who were either non-Lyme infected without a history of Lyme disease or who had recovered from acute Lyme disease. We hypothesized that patients with PTLD would have more CS impairment than controls and that this impairment would be associated with neurologic and cognitive abnormalities.

## Methods

### Study Participants

Patients with PTLD were recruited for vision testing from 2015 to 2020 within a larger cross-sectional study.^10^ Detailed eligibility criteria for this study, which uses the Infectious Diseases Society of America's case definition for PTLD,^7^ have been described elsewhere.^10^ Briefly, study participants were required to have medical record–confirmed prior Lyme disease and appropriate antibiotic treatment, as well as continued fatigue, pain, or cognitive dysfunction that affected general functioning. Participants were excluded for a range of specific, comorbid medical conditions.^10^ For the current analysis, we did not require participants to have been ill for longer than 6 months at the time of the study visit. We excluded 22 participants who self-reported a prior diagnosis of cataract or glaucoma. Only 3 participants with PTLD refused participation.

Control participants were drawn from two sample populations: those without a history of Lyme disease and those who had recovered from acute Lyme disease. Those without a history of Lyme disease were recruited from a clinic population as well as through flyers and online advertising. To be eligible, they were required to have a negative two-tier serologic test for antibodies to *B. burgdorferi* at the time of enrollment and to not have a prior medical history consistent with Lyme disease. Those who had recovered from acute Lyme disease following antibiotic treatment were enrolled in a longitudinal cohort study, as previously described.^22^ They were required to meet a definition for having returned to health at both the 6-month and 1-year follow-up time points.^23^ CS testing was performed for these control participants at the 6-month follow-up study visit.

All control participants were excluded for the same comorbid conditions as were patients with PTLD. One control participant was excluded for cataract, and no control participants refused participation.

The Institutional Review Board of the Johns Hopkins University School of Medicine approved this study. Written informed consent was obtained from all study participants prior to initiation of study activities. This study was performed in accordance with the Declaration of Helsinki.

### Visual Testing

CS was tested using a Pelli–Robson CS chart, which consists of eight rows of two triplets per row and a range in contrast from 0.00 to 2.25 logCS.^24^ The change in contrast is 0.15 log units between triplets. Each eye was tested separately at 1 m using a different chart and the participant's usual eyewear. Forced-choice procedures were used, in which participants had to identify two of the three letters in each triplet to proceed with testing. The total number of letters read correctly (CS score) was recorded for each eye and converted to logCS. One technician tested all but 12 of the study participants, and all but 4 were tested using the same standardized room; the 4 were tested using the same charts and procedures but in a different room with the same lighting characteristics. CS impairment was defined using cutoffs appropriate for age less than 60 years as logCS of 1.80 (36 letters) and for those equal to or over age 60 years as logCS of 1.65 (33 letters).^25^

The Early Treatment for Diabetic Retinopathy Study charts and protocols were used for visual acuity testing,^26^ and each eye was tested separately using a different chart. Forced-choice procedures were used, with at least four of the five letters in each line required to be read correctly for testing to proceed. The number of letters read correctly was recorded, converted to Snellen equivalent for analyses, and categorized into the following for analysis: better than 20/40 in both eyes, worse eye between 20/40 and 20/70, and worse eye worse than 20/70.

A detailed ophthalmic examination was not performed.

### Clinical Interview, Neurologic Exam, and Cognitive Testing

All participants completed the Post-Lyme Questionnaire of Symptoms (PLQS) that inquired about the presence and severity of 36 symptoms over the past 2 weeks (0 = absent, 1 = mild, 2 = moderate, or 3 = severe). The ocular symptoms “double vision,” “eyes sensitive to light,” and “changes in vision clarity” were stratified into present (moderate or severe) or absent (absent or mild), and the resulting binary variables were included in this analysis.

Participants underwent a physical exam that included specific tests of neurologic function (recorded as normal or abnormal): examination of cranial nerves 2 to 12, motor strength, reflexes, coordination, and gait, as well as a sensory exam including measurement of pain and light touch as well as proprioception. Vibratory index was measured on the distal interphalangeal joint of the index finger and on the interphalangeal joint of the hallux using a Rydel–Seiffer 64-Hz tuning fork.^27^ Since so few abnormalities on these tests were found within the control sample, the summary variable “any neurologic sign” (if any one of these tests were abnormal) was used to evaluate group differences.

Lastly, a battery of cognitive tests was conducted.^28^ Eight T-scores were generated across the following cognitive tests: the Digit Span and the Coding subtests of the Wechsler Adult Intelligence Scale–Fourth Edition,^29^ the Hopkins Verbal Learning Test–Revised (total recall, delayed recall, retention, and recognition discrimination),^30^ and the Trail Making Test parts A and B.^31^ An estimate of premorbid intellectual functioning was measured using the Wide Range Achievement Test–Fourth Edition, reading subtest.^32^ A participant was considered cognitively impaired if at least 2 of the 8 cognitive tests’ T-scores were more than two standard deviations below both (a) the population mean (i.e., below a cutoff score of 30) and (b) the participant's premorbid intellectual ability T-score. In addition, impairment was independently assessed for Trails A and B to specifically examine tests of attention.

### Statistical Analyses

First, we tested for differences between participants with PTLD and controls using bivariate analyses, then a logistic regression model. The associations between CS impairment and other factors were assessed within the PTLD group through multiple logistic regression models, adjusting for age and race. *P* < 0.05 was considered statistically significant. Due to quasi-complete separation in the data for one cognitive test, a penalized likelihood estimation method (Firth method) was used.

## Results

Participants with PTLD all met CDC criteria for prior “confirmed” or “probable” Lyme disease,^33^ and 97.2% were residents of states with a high incidence of Lyme disease at the onset of their illness.^34^ Participants had been ill with PTLD a median of 1.5 years (interquartile range, 0.60–3.79; range, 0.06–28.59) and 40.4% were two-tier seropositive for antibodies to *Borrelia burgdorferi* at the time of enrollment. There was no difference by age, race, gender, or visual acuity between cases and controls (Table 1). Participants with PTLD were statistically significantly more likely than controls to meet criteria for impairment on cognitive testing and to have ocular complaints. Only one control (1.7%) had an ocular complaint of light sensitivity, compared to 23% of cases. Although the cases and controls had similar ranges and medians of contrast sensitivity, the cases had a greater proportion that fell into the “impaired” range compared to controls (Fig.).

**Table 1.** Demographic, Ocular, Neurologic, and Cognitive Characteristics of Patients With Posttreatment Lyme Disease and Controls

Characteristic	Control (n = 60)	PTLD (n = 214)	*P* Value^a^
Demographics			
Age^b^	43.50 [34.75, 61.25] (20.00–72.00)	45.50 [36.00, 55.00] (18.00–77.00)	0.455
Male gender	30 (50.0)	119 (55.6)	0.533
White, non-Hispanic	48 (80.0)	186/213 (87.3)	0.221
Cognitive testing			
Impairment	0 (0.0)	33 (15.4)	< 0.001
Impairment (Trails A and B)	0 (0.0)	5 (2.3)	0.589
Impairment (Trails A)	2 (3.3)	16 (7.5)	0.379
Impairment (Trails B)	2 (3.3)	11 (5.1)	0.740
Ocular complaints			
Eyes sensitive to light	1 (1.7)	49 (22.9)	< 0.001
Change in clarity	0 (0.0)	37 (17.3)	< 0.001
Double vision	0 (0.0)	7 (3.3)	0.353
Visual acuity			
Both eyes better than 20/40	59 (98.3)	202 (94.4)	0.310
Worse eye between 20/40 and 20/70	1 (1.7)	10 (4.7)	0.465
Worse eye worse 20/70	0 (0.0)	2 (0.9)	1.000
Contrast sensitivity			
Left eye score	36.50 [36.00, 40.00] (32.00–43.00)	37.00 [36.00, 39.00] (30.00–44.00)	0.425
Right eye score	36.00 [35.00, 38.25] (30.00–41.00)	36.00 [35.00, 37.00] (29.00–41.00)	0.324
Impairment in either eye	12 (20.0)	84 (39.3)	0.009

a Comparisons between groups were conducted using Fisher exact tests for categorical variables, and *t*-tests or Wilcoxon rank sum tests for continuous variables as appropriate.

b Data from continuous variables not normally distributed are presented as median [25th percentile, 75th percentile] (range). Data from categorical variables are presented as count (%). One participant from the PTLD group was missing race (0.4% missing data).

**Figure.**
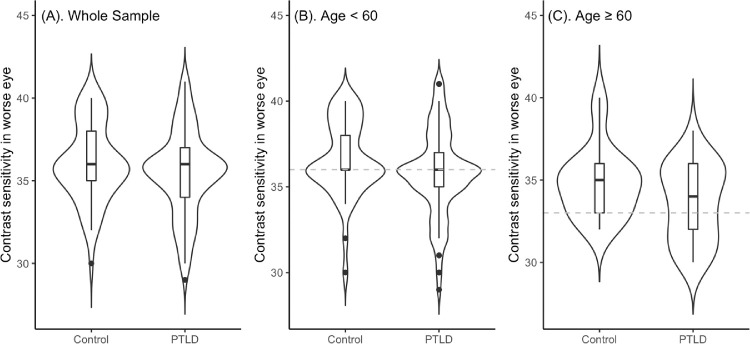
Distribution of Contrast Sensitivity in worse eye by age and case (Posttreatment Lyme Disease vs. Control) status. The cutoff for impairment is shown in the horizontal line for those age < 60 years and those ≥ 60 years.

Ninety-eight percent of controls and 94% of participants with PTLD had visual acuity 20/40 or better in both eyes, with no eyes worse than 20/100. Although CS scores in right and left eyes did not differ between participants with PTLD and controls, participants with PTLD had nearly twice the percentage of CS impairment in either eye, which was statistically significant. (Table 1). CS impairment was associated with an increased odds of being in the PTLD group that was 2.6 times as high as those without CS impairment (odds ratio, 2.6; 95% confidence interval, 1.3–5.2; *P* = 0.007).

We then examined the relationship of CS impairment with ocular complaints, neurologic abnormalities, and cognitive impairment within the PTLD group, adjusting for age and race. We did not find statistically significant associations of CS score with any of the reported ocular complaints (Table 2). In addition, the ocular complaints were not associated with CS score or worse CS score (data not shown). The odds of CS impairment were 2 to 3.6 times as high when individual neurologic exam abnormalities were present compared to when they were not. Overall, the odds of CS impairment were two times as high when any neurologic abnormality was identified compared to those with a normal neurologic exam. Similarly, the odds of CS impairment were also approximately two times as high when any impairment was identified on cognitive testing compared to those with normal cognitive testing, and this relationship was borderline statistically significant. We specifically hypothesized that impairment on tests of attention, Trails A and B, would be associated with CS impairment due to common complaints of lack of concentration and mental fogginess by patients with PTLD. In fact, this relationship was found, although few cases failed both tests. The odds of CS impairment were 4.5 times as high for cases with impairment on Trail B alone compared to those without impairment on this test, and the results were statistically significant.

**Table 2.** Separate Logistic Regression Models of the Odds of Contrast Sensitivity Impairment Among the PTLD Cases, Adjusted for Age and Race

Characteristic	Odds Ratio for CS Impairment	95% Confidence Interval	*P* Value
Ocular complaints			
Eyes sensitive to light	1.20	(0.62, 2.33)	0.584
Change in clarity	0.81	(0.39, 1.70)	0.574
Double vision	2.07	(0.44, 9.79)	0.357
Neurologic exam abnormalities			
Cranial nerve	2.62	(0.40, 17.14)	0.315
Motor	3.56	(0.67, 18.85)	0.135
Vibratory	1.96	(0.88, 4.36)	0.098
Sensory	2.45	(0.42, 14.25)	0.320
Pin	2.95	(1.07, 8.14)	0.037
Foot stand	1.95	(0.62, 6.12)	0.255
Any neurologic exam abnormality	2.06	(1.04, 4.07)	0.038
Cognitive testing			
Impairment	2.10	(0.98, 4.51)	0.058
Impairment Trails A and B^a^	17.76	(1.95, 2349.56)	0.007
Impairment Trails A	1.58	(0.56, 4.49)	0.390
Impairment Trails B	4.47	(1.1, 18.22)	0.037

Models Evaluate the Odds Associated With Individual Ocular Complaint, Neurologic Exam Abnormality, and Cognitive Testing Impairment Variables.

a Due to quasi-complete separation in the data, a penalized likelihood estimation method (Firth method) was used.

In a logistic model that included age and race, as well as both neurologic abnormality and impairment on Trail B, the strong association of CS impairment with cognitive impairment was still found, with borderline statistical significance, as well as a nonsignificant association with neurologic impairment (Table 3).

**Table 3.** Logistic Regression Model of the Odds of Impaired Contrast Sensitivity Within the PTLD Group According to Age, Race, Any neurologic exam abnormality, and Cognitive Impairment on Trials B

Characteristic	Odds Ratio	95% Confidence Interval	*P* Value
Age (10 years)	0.90	(0.71, 1.15)	0.407
Race white, non-Hispanic	1.04	(0.45, 2.39)	0.920
Any neurologic exam abnormality	1.77	(0.87, 3.60)	0.115
Cognitive impairment Trails B	3.45	(0.84, 14.23)	0.086

## Discussion

These data support a statistically significantly increased odds of CS impairment in patients with PTLD compared to controls. The patients with PTLD in this study did not report other ocular conditions, only one had any ocular findings during the acute phase of their Lyme disease, and in general, they were now found to have good visual acuity. Most case series of Lyme disease with ocular manifestations are described in Europe, where the prevailing strains of *B. burgdorferi* sensu lato are more neurotropic.^1^ These patients often have unrecognized and untreated Lyme disease at the time of evaluation; they present with visual acuity loss or blurred vision, and antibiotic treatment often resolves the visual findings.^35^^–^^44^ In one study, a high proportion of Lyme disease cases referred for evaluation had convergence insufficiency (53%), but the stage of Lyme disease was not reported and visual acuity data were not presented.^45^ In case series where ocular disease did not resolve after treatment, visual acuity loss also persisted.^41^^,^^42^^,^^46^^,^^47^ There are reports of structural changes despite acuity of 20/20 on presentation, where symptoms resolve with treatment.^48^ Because our PTLD cases had good visual acuity, and the large letter size in the Pelli–Robson chart helps ensure that refractive errors are not interfering with assessing contrast,^49^ we do not feel this difference in contrast impairment is due to overt clinical disease in the cases. However, we did not do imaging and therefore cannot rule out more subtle ocular findings related to PTLD that might be associated with these changes.

The higher rate of nonspecific ocular symptoms in participants with PTLD compared to controls has been previously reported in this sample,^10^ and we expected to see associations between these complaints and CS impairment or CS score. However, these associations were not found. There was no association of ocular complaints with acuity either, and over 90% of PTLD cases had good visual acuity. We did not do refractive correction in this study, and potentially very few could have improved on testing vision with refractive correction.

We found independent associations of CS score with neurologic abnormalities on physical exam. While possibly indicative of an abnormality in central nervous system processing, we cannot rule out subtle neurologic retinal changes. Research from other, well-characterized neurologic diseases like MS may be instructive, as CS loss in patients with MS has been well described and proposed as a visual outcome measure in clinical trials.^18^ In MS, injury anywhere along the visual pathway may produce a loss of contrast. Fisher et al.^21^ have shown that even in the absence of optic neuritis, patients with MS and depressed CS have a thinning of the retinal nerve fiber layer (RNFL) demonstrable on optical coherence tomography (OCT). CS depression is also associated with lesions in the posterior visual pathway as determined by magnetic resonance imaging.^50^ There are no comparable data examining possible lesions along the visual pathways of patients with PTLD, and further investigations would be instructive.

We also found an association between lower CS score and cognitive loss, particularly in tests of attention. We hypothesized this would be the case because of the fatigue associated with PTLD and the sustained attention required to detect the lower contrast letters. Both fatigue and difficulty focusing are among the most common complaints among patients with PTLD.^10^ Cognitive tests, particularly those that measure attention or executive functioning, as well as CS testing with forced-choice procedures, require a degree of concentration that may be difficult for these patients.^51^ Thus, this association may not reflect a problem with vision but rather the ability of PTLD cases to complete the test. There is precedence for this assumption. Depressed CS scores were found to be associated with tests of cognitive function in patients with MS, even after controlling for changes in RNFL thickness.^17^ In both cross-sectional and longitudinal associations, Ward et al.^20^ and others have found that loss of CS predicted the onset of cognitive impairment and dementia.^52^ More recent data suggest that these patients may also have retinal ganglion cell loss and optic nerve head thinning. Testing fatigue may be a common factor in the association of lower CS with lower cognitive testing scores, both for patients with PTLD and those with MS. Testing of saccadic eye movements, which can reveal more uneven patterns with decreasing levels of concentration, would have provided another measure of concentration ability and might be useful for future studies.^51^ The multivariable model that evaluated both neurologic abnormality and impaired tests of attention as factors for impaired contrast sensitivity found slightly lower risks for each variable and less statistical significance, which could suggest collinearity between the neurologic and cognitive abnormalities. More cases had neurologic abnormalities (30%) than impairment on the test of attention (5%), suggesting the stressors on neurologic function from PTLD may affect cognitive function as well.

Despite finding an association between lower CS and both neurologic abnormalities and cognitive loss, our study does not allow us to speculate on the localization of the origin(s) of decreased CS in the visual system or on the likelihood of fatigue as an explanation for the association with cognitive loss. We did not use OCT to determine the thickness of retinal layers or ascertain other potential abnormalities that may affect CS but not visual acuity. The use of functional or multifocal electroretinography testing in future studies may help differentiate the location of any structural abnormality.

This study does have limitations. We had a smaller sample of controls, and very few had any neurologic or cognitive deficits. The fact that some controls had recovered from acute Lyme disease and were now showing normal results highlights the deficits of those who do not recover and go on to PTLD. Second, we chose a CS test that was easy to administer, given that the parent study was part of a much larger, time-consuming protocol. The Pelli–Robson test measures CS only in the spatial frequency band where CS is peak; therefore, it is possible that we missed detection of deficits in higher spatial frequencies, or “notch” losses. The finding that visual acuity was largely normal in these patients provides reassurance that we were able to detect CS differences between cases with PTLD and controls that were not due to acuity differences.

The study has several strengths, including the use of a large, well-characterized cohort of cases with PTLD linked to prior infection with *B. burgdorferi*. Furthermore, the detailed neurologic and cognitive testing enabled us to evaluate associations with CS that mimic findings from other well-established disease entities. This association suggests that further investigations into patients with PTLD, who may have subtle ocular changes or other reasons for impaired CS, would prove fruitful.

In summary, we found a relationship between CS impairment in participants with PTLD compared to controls that is associated with neurologic abnormalities and specific cognitive test impairments. Further research to elucidate any possible pathophysiology associated with CS loss is warranted. At this stage, it is unclear if CS testing would be a useful marker of improvement over time in these patients, suggesting a need for further longitudinal studies.
